# Health Risks Associated with Occupational Exposure to Ambient Air Pollution in Commercial Drivers: A Systematic Review

**DOI:** 10.3390/ijerph15092039

**Published:** 2018-09-18

**Authors:** Herve Lawin, Lucie Ayi Fanou, Antoine Vikkey Hinson, Marie Stolbrink, Parfait Houngbegnon, Nonvignon Marius Kedote, Benjamin Fayomi, Jacqueline Kagima, Patrick Katoto, Edgard Marius Dona Ouendo, Kevin Mortimer

**Affiliations:** 1 Unit of Teaching and Research in Occupational and Environmental Health, Faculty of Health Sciences, University of Abomey-Calavi (UAC), Cotonou 03BP0490, Benin; hinsvikkey@yahoo.fr (A.V.H.); bfayomi2@yahoo.fr (B.F.); 2 Institut Régional de Santé Publique, University of Abomey Calavi, Cotonou 01 BP 526, Benin; phoungbegnon@yahoo.fr (P.H.); kedmar@yahoo.fr (N.M.K.); eouendo@yahoo.fr (E.M.D.O.); 3 EcoHealth Chair, University of Abomey Calavi, Cotonou 01 BP 526, Benin; 4 Laboratoire de Biochimie et de Biologie Moléculaire, FAST/UAC, Cotonou 01 BP 526, Benin; afaluc@yahoo.fr; 5 Liverpool School of Tropical Medicine, Liverpool L3 5QA, UK; mstolbrink@doctors.org.uk (M.S.); jacikagima@gmail.com (J.K.); Kevin.Mortimer@lstmed.ac.uk (K.M.); 6 Department of Internal Medicine and the Hôpital Provincial General de Reference de Bukavu, Universite Catholique de Bukavu, South Kivu, Democratic Republic of the Congo; katotopatrick@gmail.com; 7 Centre for Environment and Health, Department of Public Health and Primary Care, Laboratory of pneumology, KU Leuven, 3000 Leuven, Belgium

**Keywords:** air pollution, health risk, driving, automobile, bus, motorcycle

## Abstract

Ambient air pollution is a major global health problem and commercial drivers are particularly exposed to it. As no systematic assessment of the health risks associated with occupational exposure to ambient air pollution in this population had yet been carried out, we conducted a systematic review using a protocol-driven strategy. Papers published from inception to April 20, 2018 in MEDLINE, EMBASE, CINAHL, African journals online, the Cochrane library, ISRCTN WHO ICTRP, and the Web of Science and Scopus databases were screened for inclusion by two independent reviewers. Original articles with at least an available abstract in English or French were included. The initial search retrieved 1454 published articles of which 20 articles were included. Three studies reported a significant difference in white blood cells (10^6^/L) among commercial motorcyclists compared to rural inhabitants (5.041 ± 1.209 vs. 5.900 ± 1.213, *p* = 0.001), an increased risk of lung cancer (RR = 1.6, 95%CI 1.5–1.8) in bus drivers and an increased standardized mortality ratio (SMR) in bus drivers from Hodgkin’s lymphoma (SMR 2.17, 95%CI 1.19–3.87) compared to white-collar workers. Other studies also found that drivers had more oxidative DNA damage and chromosome breaks. Four papers failed to demonstrate that the drivers were more exposed to air pollution than the controls. Three other studies also reported no significant difference in lung function parameters and respiratory symptoms. The genetic polymorphisms of detoxifying enzymes were also not homogeneously distributed compared to the controls. There is some evidence that occupational exposure to ambient air pollution among commercial drivers is associated with adverse health outcomes, but the existing literature is limited, with few studies on small sample size, methodological weaknesses, and contradictory findings—thus, further research is recommended.

## 1. Introduction

Air pollution is a major global public health problem that caused 7 million deaths in 2012, including 3.7 million due to ambient air pollution [1]. The majority (88%) of the deaths due to ambient air pollution occurred in low- and middle-income countries. Traffic air pollution is responsible for much of the ambient air pollution in cities, with exhaust emissions alone accounting for up to 30% of all particulate matter emitted in urban areas [2]. The International Agency for Research on Cancer (IARC) classified ambient air pollution (particularly particulate matter) as a group 1 carcinogen for the lungs [3]. Commercial drivers of buses, cars, and motorcycles in urban areas are commonly exposed to ambient air pollution in the course of their work. They represent an important part of the labor force in several urban areas, especially in low and middle incomes countries (LMIC) [4,5] and are occupationally exposed to air pollution. The vehicles they drive are both sources of air pollution for the drivers and others who work in the outdoor environment. Due to the severity of air pollution exposure and their large number, especially in LMIC, it becomes important to know whether their occupational exposure is a source of additional health risks compared to the general population who are not occupationally exposed to air pollution. The recognition of occupational exposure to air pollution as an occupational health risk for commercial drivers requires a robust study method, including, among others, a study population without selection bias and significant results. These key factors are important in defining whether an increased health risk is limited, or even unique, to commercial driving in relation to the occupational exposure to air pollution. The healthy worker effect may also alter the observed health effect and lead to contradictory findings. To our knowledge, there has not yet been a systematic review of the literature to clarify this question on the health risks associated with occupational exposure to ambient air pollution amongst commercial drivers. A recent review was done only on the effect of air pollutants on airways, which included all outdoor workers except commercial drivers [6]. The current review has thus set out to fill this gap with a view to identifying knowledge gaps, opportunities for further research, and to guide policies to help protect the health of this vulnerable group.

## 2. Methods

### 2.1. Data Sources and Search Strategy

We searched MEDLINE, EMBASE, CINAHL, African journals online, the Cochrane library, ISRCTN WHO ICTRP, and the Web of Science and Scopus databases for papers written in the English and French language, published from inception to April 20, 2018 by using a systematic search strategy with the removal of duplicate titles. Table 1 reports the key words that were used. Reference lists from published reviews and included publications, abstracts from major occupational and environmental medicine conference proceedings of major conferences on occupational and environmental health were also searched. The bibliography of studies included in the review were searched for any additional, relevant titles.

### 2.2. Study Selection

Original studies comparing the health effects of occupational air pollution in commercial drivers and a comparison group were included if they met the selection criteria detailed in Table 2. Two (HL, MS) authors screened the titles and abstracts and made study selection decisions independently. A third author (LAF) reviewed it, in case of disagreement. There were no study design restrictions.

### 2.3. Data Extraction, Risk of Bias Assessment and Analysis

Data was collected about the study design and the location, type, and number of drivers and their controls, the type of exposure, and outcomes measured. A narrative synthesis was completed on all the included studies and reported the key points on each of the studied items. 

## 3. Results

**Figure 1:**
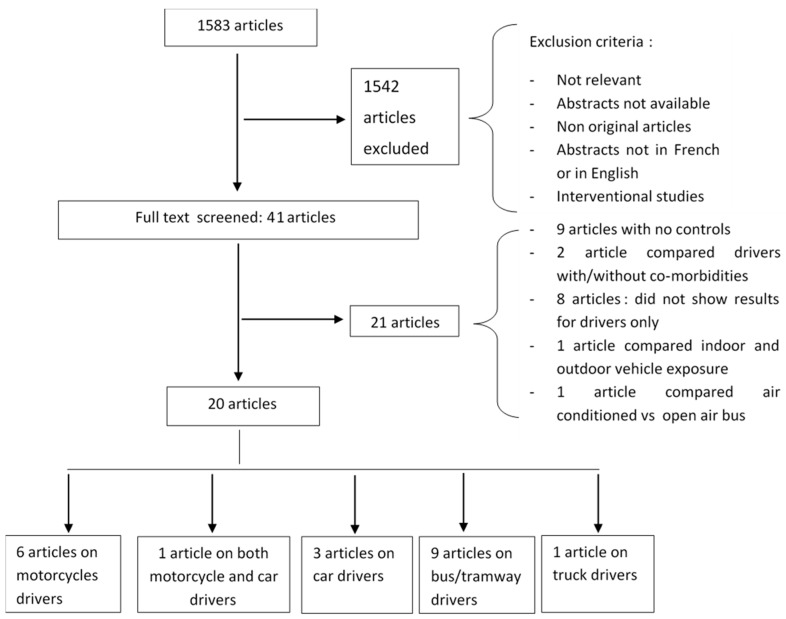
Preferred reporting items for systematic reviews and meta-analyses (PRISMA) flow diagram.

The initial search retrieved 1583 published articles, of which 1542 were excluded based on their titles and abstracts. A full evaluation of 41 papers found 20 articles relating to commercial motorcycles, including cars, buses, and trucks (Table 3, Table 4, Table 5 and Table 6). The 21 non-included papers were excluded on the basis of: nine articles having no comparative group; two papers comparing drivers with/without co-morbidities; eight articles not reporting results for drivers only; one article comparing indoor and outdoor vehicle exposure; and one article comparing air-conditioned vs. open air buses (Figure 1).

### 3.1. Study Design and Site

All the included studies were observational—17 cross-sectional, and 3 cohort. All the cohort studies were implemented in high-income countries, and almost one-third of the cross-sectional studies were done in Africa.

### 3.2. Populations Studied

Most of the articles (13 of 20) studied bus and commercial motorcycle drivers, while three articles assessed car taxi drivers. The comparison groups varied considerably between studies, such as drivers who were not occupationally exposed to ambient air pollution in the same location, rural/suburban inhabitants, administrative and office workers, policemen, or civil servants. Only two studies used age and gender which matched in the recruitment of the comparative group—one of these recruited the matched population from a rural area different to the working area of the exposed group, and the other recruited the matched comparative group from the same locality.

### 3.3. Exposure Variables Measured in Studies Included in the Review

Several types of pollutants were measured to characterize the exposure level in the drivers: particle matters; volatile organic compounds (including benzene, toluene, ethyl benzene, and xylene (BTEX)); polycyclic aromatic hydrocarbons (PAHs) (including benzo[a]pyrene (B[a]P)); and gaseous pollutants (SO_2_, CO, NO). Most of the measurements were performed by using fixed monitoring stations. These fixed stations pre-existed or were set up for the purpose of the studies. Some personal measurements were performed by using urine excretion of PAHs or benzene and by the dosage of carboxyhemoglobin. The measurement duration varied between studies, but 24 to 48 h or one week of exposure measurement duration were most frequently done. Some measurements were also performed before and after work. Two studies were used the job title alone to define the exposure.

Of the 20 studies included in this review, four found no difference in air-pollution exposures between the exposed and control groups. Brucker et al. [14] and Burgaz et al. [15] reported that exposure to carboxyhaemoglobin (almost 2% in the two groups) and 1 hydroxypyrene (0.32 ± 0.25 vs. 0.57 ± 0.36 µmol/mol creatinine, *p* > 0.05) were not different in a group of taxi drivers and controls. Brucker compared 39 automobile taxi drivers with 21 non-occupationally exposed controls, and Burgaz compared 17 taxi drivers with 23 office workers. Fanou et al. [8] also reported a statistically insignificant difference in 1-hydroxypyrene level among six urban motorcycle taxi drivers and five rural inhabitants. Rossner et al. [24] reported that bus drivers were less exposed to B[a] P (1.3 ± 0.7 vs. 1.8 ± 1.0 mg/m^3^, *p* < 0.01) and carcinogenic PAHs (7.1 ± 3.7 vs. 9.4 ± 5.5 mg/m^3^, *p* < 0.05) than the controls. The controls were healthy male volunteers spending > 90% of time indoors daily.

### 3.4. Outcome Variables Measured in Studies Included in the Review

Most of the outcomes measured were based on reactive oxygen species (ROS) that produced oxidative stress and DNA damage. The current studies reported measurement of oxidative DNA, oxidized protein and lipids, DNA adducts, and chromosome aberrations and breakage. Cytochrome P4501A1 (CYP1A1), which is the main enzyme of the metabolic activation of PAHs, was measured. Genetic polymorphisms of glutathione S transferase (GSTs), which can detoxify the carcinogenic activity of the PAHs, were also measured. Inflammatory biomarkers (cytokines, high-sensitivity C reactive protein) were also reported in a few studies. The results of these intermediate markers of health risks were contradictory. Four studies reported no difference in the distribution of these markers. Avogbe et al. [7] and Petchpoung et al. [23] reported that there was no statistical significant difference in the protective gene distribution (GST, CYP1A1, Glutathione peroxidase (GPX), NAD(P)H:quinone oxido-reductase 1) between the drivers and controls (rural and suburban residents). Although the level of chromosome break was higher among the urban taxi drivers (*n* = 30), Taghizadeh et al. [16] did not find any statistical difference (6.7% vs. 3.3%, *p* = 0.3) compared to the rural taxi drivers (*n* = 30). Bagryantseva et al. [18] reported no difference in the level of oxidative DNA damage between the drivers and administrative workers (2.35 ± 2.17 vs. 2.55 ± 2.86% of tail DNA damage, *p* > 0.05).

Besides these intermediate outcomes, clinical endpoints were also measured. Lung function parameters, standardized mortality rate, ischemic heart disease mortality, as well as blood-cell count were reported. Three studies failed to demonstrate that the commercial drivers had more clinical health risks than their controls. Comparing motorcycle taxi drivers (*n* = 85) and an individual matched control group in Cotonou (Benin), Lawin et al. [13] reported no difference in the prevalence of cough and/or phlegm (adjusted odds ratio (AOR) 1.57, 95% confidence interval (CI) 0.51–4.84) and in lung-function parameters (adjusted difference in forced expiratory volume in 1 s (FEV1) 0.12L, 95% CI −0.16–0.22; adjusted difference in forced vital capacity (FVC) 0.11, 95% CI −0.14–0.37). In the same area, Fourn et al. [11] also reported no difference in respiratory symptoms between drivers (*n* = 250) and non-drivers in Cotonou (*n* = 150) (odds ratio 1.18, 95% CI 0.70–2.00). Ekpenyong et al. [10] in Uyo metropolis (Nigeria) reported a higher frequency of lung function disorders by comparing commercial motorcyclists (*n* = 24) to six civil servants (FEV1 < 80% predicted AOR 1.01, 95% CI 0.942–1.081; FVC < 80% predicted AOR 3.10, 95% CI 0.402–16.207) and car taxi drivers (*n* = 18) to the same civil servants (FEV1 < 80% predicted AOR 1.02, 95% CI 0.953–1.091; FVC < 80% predicted AOR 1.72, 95% CI 0.408–4.732) although the difference was not statistically significant.

Four studies showed evidence of clinical health risks associated with occupational exposure to ambient air pollution in drivers. Avogbe et al. [12] reported a significant difference in white blood cells (10^6^/L) among commercial motorcyclists in Cotonou (*n* = 144) compared to 30 rural inhabitants (5.041 ± 1.209 vs. 5.900 ± 1.213, *p* = 0.001). Soll-Johanning et al. [26] found that bus drivers in Copenhagen had an increased risk of lung cancer (relative risk (RR) 1.6, 95% CI 1.5–1.8) and bladder cancer (RR 1.4, 95% CI 1.2–1.6). Merlo et al. [21] also found an increased standardized mortality ratio (SMR) in bus drivers (*n* = 6510) from Hodgkin’s lymphoma (SMR 2.17, 95% CI 1.19–3.87) and lung cancer (SMR 1.16, 95% CI 1.05–1.28) compared to white-collar workers (*n* = 601) in Italy. This risk mortality increased after 30 years of employment. Hart et al. [17] also reported an increased risk of mortality from ischemic heart disease associated with at least one year of work for truck drivers in the U.S. (hazard ratio 1.44, 95% CI 1.22–1.70).

## 4. Discussion

This study was the first systematic review to assess the health risks associated with occupational exposure to ambient air pollution in commercial drivers. Despite the increasing number of people in this particular occupation, we were able to find only a few studies on this topic, especially in LMIC. Most of the articles focused on bus and motorcycle taxi drivers, which represent the main methods of public transport in LMIC, especially in Africa [13]. These methods of transport, especially in African settings, contribute substantially to the level of air pollution in urban cities due to the age of vehicles and fuel quality [27]. Four studies reported that commercial drivers had decreased white blood cell counts [12], increased risks for lung and bladder cancer [26], as well as increased risks of mortality from Hodgkin’s lymphoma, lung cancer [21], and ischemic heart disease [17]. Oxidative DNA damage, DNA adducts and strand breaks, and chromosome aberration that was found in these drivers may help to explain the increased risk of cancer.

However, there was a wide variation in the methods and endpoints of the assessed studies. Seven studies found no significant differences between drivers and controls. Hence, we were unable to definitively conclude that the health effects reported in drivers were fully attributable to occupational exposure to ambient air pollution.

The main methodological weaknesses of the included studies were related to the choice of the comparative study population. In most of the studies, drivers were compared to rural/suburban inhabitants, administrative and office workers, policemen, civil servants, or other drivers in rural areas. As such, the control groups were not always appropriate, as they would also have experienced considerable air pollution exposures—for example, to household air pollution—and the lack of adjustment for such exposures may have contributed to some of the contradictory findings seen between studies.

Four studies failed to demonstrate that drivers were more at risk of exposure than the controls. For three studies [8,14,15], this is possibly due to a lack of statistical power, given their small sample sizes. The fourth study [24] found that controls were more exposed to B [a] P and carcinogenic PAHs than bus drivers. One possible explanation for this is that the use of closed bus-driver cabins with closed windows may have reduced their exposure, hence the small variation in the air pollution exposure due to a specific job activity. The use of a fixed station without a land-use regression model [28] may not, then, adequately characterize individual exposures.

Three of the cross-sectional studies found no difference in respiratory outcomes between drivers and controls [10,11,13]. These contradictory results can be explained by the choice of controls and/or the lack of statistical power, as noted above. The use of convenience sampling for both drivers and controls and a lack of control over confounding variables further explains the contradiction in these studies. The healthy-worker effect commonly found in cross-sectional studies can also explain these results.

Taghizadeh et al. [16] and Bagryantseva et al. [18] also reported no difference in the frequency of the chromosome breaks and DNA damage compared to controls. The genetic polymorphisms of the detoxifying enzymes, their metabolic activation, and their distribution in the population can also explain these contradictory results. These genes act as modulators or effect modifiers. GST and CYP1A1 contributed in the metabolic activation of the detoxification of the carcinogenic PAHs. Avogbe et al. [7] and Petchpoung et al. [23] reported that these genes were not homogeneously distributed in drivers and controls and may not be activated in low air-pollution exposure, especially in low PAH exposure.

There is a need to carry out studies with robust methods to define whether commercial driving is a risky job in relation to the occupational exposure to ambient air pollution. Intermediate health outcomes, such as the genetic polymorphism of GST, GPX, and CYP1A1 may be considered in the assessment of clinical health risks. Although they do seem to modify short-term clinical health risks, their detoxification ability may be altered in the long term. This reduction of their ability may be associated with the health risks (cancer risks, increased mortality) that were reported in the cohort studies among drivers in Denmark, the U.S., and Italy [17,21,26]. 

## 5. Conclusions

In the present study, we were able to find some evidence that occupational exposure to ambient air pollution among commercial drivers is associated with adverse health outcomes, but the existing literature is limited as there are only a few studies available with small sample sizes, methodological weaknesses, and contradictory findings. We recommend that future research should have more robust methods and consider the distribution of genetic polymorphisms. At the same time, there is evidence that exposure to air pollution is harmful to human health with established clean air interventions (including clean air legislation). Alongside further research in this area, we recommend that effective interventions for purifying the air are implemented for all.

## Figures and Tables

**Table 1:** Keywords.

MeSH* Keywords		
- “Motorcycle” - “Motorbike” - “Automobile Driving” - “Taxi driver” - “Professional driver” - “Bus driver” - “worker” - “Commerce” - “Transit worker”	**And**	“Air pollution”

MeSH = Medical Subject Headings.

**Table 2:** Selection criteria.

Inclusion Criteria	Exclusion Criteria
Original articles Focus on air pollution and drivers Abstracts available Abstract available in English, French Study population must include at least one comparative group	In vitro Interventional studies Studies on animal, cyclists or walkers

**Table 3:** Studies on motorcycle taxi drivers included in the review.

Authors	Study Design/Site	Type of Drivers (Number)	Comparative Study Population (Number)	Exposure	Outcomes	Key Findings
Avogbe et al. [7]	Cross-sectional/Cotonou (Benin)	Motorcycle (*n* = 29)	1. Rural subjects (*n* = 27) 2. Roadside residents (*n* = 37) 3. Suburban subjects (*n* = 42)	1. PM0.1 (fixed site) measured during the working day 2. S-phenylmercapturic acid (S-PMA)	1. oxidative DNA damage in mononuclear blood cells: strand breaks (SB) and formamidopyrimidine glycosylase (FPG) 2. GlutathioneS-transferase (GST) 3. Glutathione peroxidase (GPX) 4. NAD(P)H:quinone oxidoreductase 1 (NQO1)	1. Stepwise exposure gradient (rural Subjects < suburban subjects < Roadside residents < taxi-moto drivers) 2. NSD in the distribution of most of the genes + inhomogeneous distribution 3. SD in the distribution of SB and FPG sensitive sites
Ayi Fanou et al. [8]	Panel study/Cotonou (Benin)	Motorcycle stage 1 (*n* = 35) stage 2 (*n* = 6)	Stage 1 1. Rural subjects (*n* = 6) Stage 2 1. Rural subjects (*n* = 5) 2. Roadside residents (*n* = 12)	1. Urine benzene 2. S-PMA 3. 1-hydroxypyrene (1-OHP)4. Personal exposure to Benzene, Toluene, Ethyl benzene and Xylene (BTEX) per week	1. DNA adducts 2. DNA fragmentation 3. oxidized DNA: 8-hydroxy-2V-deoxyguanosine (8-oxodG) and 5-methylcytosine (m5dC)	1. More BTEX and S-PMA in urban drivers than in rural residents 2. NSD of BTEX and S-PMA difference in taxi-drivers and roadside residents 3. NSD of 1-hydroxypyrene (urban drivers vs. rural area) 4. SD in DNA damage (when compared urban drivers vs. rural area inhabitants) but NSD in oxidized DNA (when comparing urban drivers vs. rural area inhabitants)
Ayi Fanou et al. [9]	Cross-sectional/Cotonou (Benin)	Motorcycle (*n* = 13)	1. Street vendors (*n* = 16) 2. Gasoline sellers (*n* = 17) 3. Roadside residents(*n* = 11) 4. Suburban residents(*n* = 20) 5. Rural inhabitants	1. Benzene (fixed site)/working day 2. Polycyclic aromatic hydrocarbons (PAHs)mainly benzo(a)pyrene (B[a]P) (6h/day/3 consecutive days) 4. 1-OHP 5. Phenol (urine)	DNA adducts	1. urban drivers are more exposed than rural inhabitants 2. NSD in Phenol and 1-OH level among urban drivers, street vendors, gasoline sellers vs. roadside residents 3. More DNA adducts in urban drivers than rural inhabitants
Ekpenyong, Ettebong et al. [10] *	Cross-sectional/Uyo metropolis, (South-South Nigeria)	Motorcycle (*n* = 24) Automobile taxi (*n* = 18)	Civil servants (*n* = 6)	1. CO 2. SO_2_ 3. NO_2_ 4. PM2.5 and PM10 Fixed station /07:30 and 09:30 (peak traffic periods) and 15:30 to 17: 30 (low traffic periods) and some personal exposure	1.Respiratory symptoms 2. Lung function	1. NSD in lung function impairment in drivers vs. civil servants 2. More respiratory symptoms among drivers
Fourn and Fayomi [11]	Cross-sectional/Cotonou and Lokossa (Benin)	Motorcycle (*n* = 250 in Cotonou *n* = 150 in Lokossa)	Non-drivers in each location	1. Personal Carboxyhaemoglobin 2. CO/morning and afternoon/Fixed station 3. Benzène/morning/Fixed station	Health disorders (headache, arterial hypertension, respiratory symptoms, digestive disorders, conjunctival hyperemia, photophobia)	1. More health disorders in Cotonou drivers 2. NSD for most of the health disorders especially respiratory symptoms (Drivers vs. non-drivers in Cotonou)
Avogbe et al. [12]	Cross-sectional/Cotonou (Benin)	Motorcycle (*n* = 144)	“Age and sex matched” Rural inhabitants (*n* = 30)	1. Benzene (personal) 3. BTEX	12 parameters from complete blood counts: total white blood cells (WBC) with four WBCsubtypes (neutrophils, eosinophils, monocytes, and lymphocytes), total red blood cells (RBC) with five red cell-related measures (hemoglobin, hematocrit, mean corpuscular volume (MCV), mean corpuscular hemoglobin concentration (MCHC) and mean corpuscular hemoglobin (MCH)) and platelets	1. Drivers were more exposed than rural inhabitants 2. Decrease only in white blood cells, lymphocyte and eosinophil counts
Lawin et al. [13]	Cross-sectional/Cotonou (Benin)	Motorcycle (*n* = 85)	Individual matched group (*n* = 85)	CO	Lung function	1. Drivers were more exposed 2. NSD in lung function and respiratory symptoms

* Study on both motorcycle and car taxi drivers. SD = statistical difference; NSD = no significant statistical difference. PM = particulate matter; DNA = deoxyribonucleic acid. CO = carbon monoxide; SO_2_ = sulfur dioxide; NO_2_ = nitrogen dioxide; BTEX = benzene, toluene, ethylbenzene, and xylene.

**Table 4:** Studies on car taxi drivers included in the review.

Authors	Study Design/Site	Type of Drivers (Number)	Comparative Study Population (Number)	Exposure	Outcomes	Key Findings
Brucker et al. [14]	Cross-sectional/Porto Alegre, Brazil	Automobile taxi (*n* = 39)	Non-occupationally exposed (*n* = 21)	1. Carboxyhaemoglobin (COHb) 2. 1-hydroxypyrene (1-OHP)	1. Platelets 2. Glucose (mg dL−1)3. Total cholesterol, HDL cholesterol, LDL cholesterol, Total cholesterol/HDL-c ratio, Triglycerides 3. Oxidized-LDL (Ox-LDL) and autoantibodies against ox-LDL (Ox-LDL-Ab) 4. Malondialdehyde (MDA) 5. Protein carbonyl (PCO) 6. Catalase (CAT) 7. Glutathione peroxidase (GPX) 8. GST 9. High-sensitivity C reactive protein (hs-CRP) 10. Homocysteine(Hcy) 11. Cytokines: Interleukin-1β (IL-1β), IL-6, IL-10,tumour necrosis factor-α (TNF-α), interferon-γ (IFN-γ) 12. Vitamin C	1. More 1-OHP in drivers than in controls but not for COHb 2. NSD for platelets, glucose, total cholesterol 3. More ox-LDL and Ox-LDL-Ab, cytokines,hs-CRP,MDA, PCO in drivers than in controls 4. Decrease in CAT, GPX, GST, vitamin C among drivers
Burgaz et al. [15]	Cross-sectional/Ankara (Turkey)	Automobile drivers (*n* = 7)	Traffic policemen (*n* = 5) Office workers (*n* = 9)	1-hydroxypyrene (1-OHP)	Chromosomal aberration (CA)	1. Controls excreted more 1-OHP than drivers and traffic policemen 2. Drivers had more CA
Taghizadeh et al. [16]	Cross-sectional/Teheran (Iran)	Urban taxi (*n* = 30)	Rural taxi drivers (*n* = 30)	N/A	1. Chromosome breakage (CB) 2. Chromosome aberration (CA) rate (including both chromosome andchromatid gaps)	1. Urban drivers had more CA 2. NSD in urban vs rural drivers regarding CB

SD = statistical difference; NSD = no significant statistical difference; N/A = not available; HDL = high-density lipoprotein; LDL = low-density lipoprotein.

**Table 5:** Study on truck drivers included in the review.

Authors	Study Design/Site	Type of Drivers (Number)	Comparative Study Population (Number)	Exposure	Outcomes	Key Findings
Hart et al. [17]	Cohort study (1985–2000)/US	Long haul up (*n* = 13,752) and Pick- and delivery (P&D) drivers (*n* = 8930)	Non-drivers in trucking industry	Job title and residential exposure to PM10, NO_2_ and SO_2_	Ischemic heart Disease (IHD) deaths (number and Hazard ratios for IHD mortality associated with at least one year of work in each specific job category)	Long haul drivers had more IHD deaths Hazard ratio = 1.44 [1.22, 1.70]

**Table 6:** Studies on bus drivers included in the review.

Authors	Study Design/Site	Type of Drivers (Number)	Comparative Study Population (Number)	Exposure	Outcomes	Key Findings
Bagryantseva et al. [18]	Cross-sectional/Prague (Czech Republic)	Bus (*n* = 50)	1. Garagemen (*n* = 20) 2. Administrative workers (*n* = 50)	1. total carcinogenic PAHs including B[a]P)/48 h 2. BTEX/24 h	1. Percentage of DNA in the tail (Tail DNA %). 2. Total DNA damage (with enzymes) 3. DNA-SB or unspecified DNAdamage; without enzymes) 3. urinary excretion of 8-oxodG 4. Urinary 15-F2t-IsoP (oxidative damage to lipids) 5. Protein carbonyl 6. Polymorphisms of metabolic genes (CYP1A1, GSTM1, GSTP1, GSTT1, EPHX3,4), folic acid metabolism genes (MS, MTHFR) and DNA repair genes (XRCC1, XPD6, XPD23, hOGG1)	1. Drivers were more exposed than administrative workers 2. Almost the same exposure for drivers and garagemen (*p* value not shown) 3. NSD in Tail DNA% (drivers vs administrative workers) 4. Drivers had more DNA-SB, 8-oxodG, 15-F2t-IsoP than administrative workers 5. Almost the same oxidative damage (drivers vs. garagemen, *p* value not shown)
Han et al. [19]	Cross-sectional/Taiwan	Bus (*n* = 120)	Office workers (*n* = 58)	N/A	8-oxodG (24 h sampling)	drivers > office workers
Hansen et al. [20]	Cross-sectional (Denmark)	Bus (*n* = 60)	Mail carriers (*n* = 88)	1-hydroxypyrene (working day and day off)	N-acetyltransferase (NAT2) phenotype	Drivers were more exposed than mail carriers
Merlo et al. [21]	Cohort study/Genoa (Italy) 1970–2005	Bus (*n* = 6510)	1.Maintenance workers (*n* = 2073) 2. White collar (*n* = 601)	Job title	Standardized mortality ratios (SMRs)	More SMRs for all causes of deaths and lung diseases in maintenance workers than in drivers than in white collar
Nielsen et al. [22]	Cross-sectional/Copenhagen (Denmark)	Bus (*n* = 90) Divided regarding gradient of exposition (central, dormitory and suburban	Rural inhabitants (*n* = 60)	N/A	DNA adducts	Drivers had more DNA adducts
Petchpoung et al. [23]	Cross-sectional/Bangkok (Thailand)	Bus (*n* = 100)	Rural inhabitants (*n* = 100)	1-OHP	1. cytochrome P4501A1 (CYP1A1) 2. GSTM1 3. GSTP1 4. GSTT1	1. Driver excreted more 1-hydroxypyrene (1-OHP) 2. The genotypedistribution was almost the same
Rossner et al. [24]	Cross-sectional/Prague (Czech Republic)	Bus (*n* = 50)	controls (*n* = 50) healthy male volunteers spending >90% of daily time indoors	PM 2.5 PM 10 cPAHs (B[a]P)	1. PCO 2. 8-oxodG 3. 15-F2t-IsoP 4. Nitrotyrosine (NT)	1. cPAHs: controls > drivers 2. More oxidative stress in drivers
Rossner et al. [25]	Cohort/Prague (Czech Republic) 03 seasons	Bus (*n* = 50)	controls (*n* = 50) healthy male volunteers spending >90% of daily time indoors	PM 2.5 PM 10 cPAHs (B[a]P)BTEX Personal/fixed monitoring	1. PCO 2. 15-F2t-IsoP	PCO and 15-F2t-IsoP: Drivers > controls in both winter (2005–2006) but not in summer
Soll-Johanning et al. [26]	Cohort/Copenhagen (Denmark)	Bus (*n* = 18,120)	Other people in Denmark	Job title	Cancer risk	Drivers > general population Lung cancer rates [relative risk (RR) = 1.695% confidence interval (95% CI) = 1.5–1.8] and bladder cancer rates (RR = 1.4, 95% CI = 1.2–1.6)

N/A = not available; PM = particulate matter.
